# Ethanolic Extract of *Butea monosperma* Leaves Elevate Blood Insulin Level in Type 2 Diabetic Rats, Stimulate Insulin Secretion in Isolated Rat Islets, and Enhance Hepatic Glycogen Formation

**DOI:** 10.1155/2014/356290

**Published:** 2014-03-31

**Authors:** Mehdi Bin Samad, Ashraf Ul Kabir, Ninadh Malrina D'Costa, Farjana Akhter, Arif Ahmed, Mohammad Rajib Jahan, J. M. A. Hannan

**Affiliations:** Department of Pharmacy, North South University, Plot 15, Block B, Bashundhara R/A, Dhaka 1229, Bangladesh

## Abstract

We measured a vast range of parameters, in an attempt to further elucidate previously claimed antihyperglycemic activity of *Butea monosperma*. Our study clearly negates the possibility of antidiabetic activity by inhibited gastrointestinal enzyme action or by reduced glucose absorption. Reduction of fasting and postprandial glucose level was reconfirmed (*P* < 0.05). Improved serum lipid profile via reduced low density lipoprotein (LDL), cholesterol, triglycerides (TG), and increased high density lipoprotein (HDL) was also reestablished (*P* < 0.05). Significant insulin secretagogue activity of *B. monosperma* was found in serum insulin assay of *B. monosperma* treated type 2 diabetic rats (*P* < 0.01). This was further ascertained by our study on insulin secretion on isolated rat islets (*P* < 0.05). Improved sensitivity of glucose was shown by the significant increase in hepatic glycogen deposition (*P* < 0.05). Hence, we concluded that antihyperglycemic activity of *B. monosperma* was mediated by enhanced insulin secretion and enhanced glycogen formation in the liver.

## 1. Introduction

Throughout the recorded history, diabetes mellitus has affected the life of millions of people of all age, sex, race, and economic status [1–4]. Studies show that, between 2009 and 2034, the number of people with diagnosed and undiagnosed diabetes will increase from 23.7 million to 44.1 million in the United States [5]. Throughout the world, diabetes mellitus is a significant reason for hospitalization, disability and in consequence imposes great financial losses to the society [6]. Epidemiologic studies and randomized clinical trials indicate that type 2 diabetes is somewhat preventable through diet and lifestyle modifications [7]. However, majority would still require taking hypoglycemic agents [8]. These agents are highly effective; however, their use is still restricted due to their high cost, adverse effects, high incidences of secondary failure, and extremely limited pharmacokinetic properties [9]. Plants have been a source of medicinal treatments for eons, and phytotherapy continue to play an indispensible role in primary health care of about 80% of the world's underdeveloped and developing countries [10]. The trend of using complementary and alternative medicines (CAM) has increased in recent years both in the developing and developed countries [11]. Due to limited research, findings on proving safety and efficacy of CAM are scarce [12]. Hence, time demands pharmacological profiling of such drugs in order to indentify the ones that are truly effective. Antidiabetic drugs work in one of the following ways: enhancement of insulin secretion, decreased production of glucose from glycogen, increased insulin sensitivity, reduced carbohydrate absorption, and increased cell utilization of cellular glucose [9]. Our present studies elucidate the basic mechanisms of antidiabetic action of* Butea monosperma*.* B. monosperma* has been previously studied for its antidiabetic activity. Antihyperglycemic activity of* B. monosperma* has been shown in type 2 diabetic rats [13–16]. Studies have found improved serum HDL level, reduced cholesterol, and LDL levels [13–16]. Its protective role on liver and pancreas, due to its antioxidative activity has also been widely studied [15–17]. Sharma and Garg have shown in vivo antioxidant effect, hypolipidemic property, and antidiabetic effect on leaves of* B. monosperma* and provide a sound foundation of this present study [18]. However, no study has been undertaken to elucidate the principal mechanism of its antihyperglycemic activity. Toxicity study (unpublished data) shows* Butea monosperma* to be safe up to a dose of 2000 mg/kg. Our study has been systematically organized to reconfirm the above claims and study the principal mechanism in which* B. monosperma* might function. To assay the potential insulin secretagogue activity, we have employed in vivo assay of serum insulin of type 2 diabetic model rats and isolated rat Islets. Liver glycogen content was assayed, in tandem, with the above studies. Soluble dietary fibers present in certain plants known for their antidiabetic activity are capable of binding with glucose, thus retarding glucose absorption [19]. Effect of* B. monosperma* on glucose absorption and sucrose digestion was analyzed employing a wide array of in vitro and in situ techniques. The objective of our current study is to elucidate the pancreatic and extrapancreatic activity of* B. monosperma.*

## 2. Materials and Methods

### 2.1. Plant Collection and Processing

Leaf of* B. monosperma* was procured from Sonpara village of Narshindhi District, Bangladesh. The plant was identified by a botanist prior to further processing and a voucher specimen (specimen number: BG-234/E3) was deposited at the National Hebarium, Dhaka, Bangladesh. The leaves were cleaned off of dirt and other debris and then thoroughly washed under running tap water to reduce the microbial contamination load. They were then air dried in an oven at 40°C and milled into a fine powder. 100 gm of this powder was then dissolved in 1 L ethanol and shaken in an orbital shaker (550 rpm for 48 hr). The mixture was then cloth filtered to remove the coarse insoluble particles. The fine particles were forced to sediment by centrifugation (1500 rpm for 10 min). The supernatant was carefully pipetted out and was further filtered using a Whatman filter paper. The filtrate was then concentrated by vacuum evaporation at 75°C using the Soxhlet apparatus (Electrothermal Soxhlet Extractor, UK). The concentrate was left in a refrigerator for 7 days to remove ethanol further, converting it into a gummy substance. This then underwent freeze drying at −55°C to obtain a fine powder. The fine powder extract was kept in Scott bottles along with silica gel sachets (desiccant) until further use.

### 2.2. Animal Handling

Both the healthy and type 2 diabetic rats (Long Evan type) were bred in the animal house of the Department of Pharmacy Practice, North South University, Dhaka, Bangladesh. The healthy rats weighted about 180–220 gm, while the type 2 diabetic rats weighed about 160–180 gm at the time of the experiment. All test animals were kept in the North South University animal house at an ambient temperature of 22 ± 5°C and humidity of 50–70%. 12 hr day-night cycle was maintained to avoid fluctuations in the circadian rhythm. Standard rat pellets (collected from ICDDR, B) and filtered drinking water were made available to the test animals ad libitum throughout the experiment apart from the period of fasting prior to certain tests. During fasting only water was given. During most experimental period, the rats were kept in translucent plastic cages with wood shavings provided as bedding. Animals undergoing fasting were placed in grilled bottomed cages, with no bedding, to prevent coprophagy. The designed experimental protocol was evaluated and subsequently approved by the Ethics Committee on Animal Research, North South University, following the “revised guide for the care and use of laboratory animals by American Physiological Society” [20].

### 2.3. Diabetes Induction

Type 2 diabetes was induced in the rats by an intraperitoneal injection of streptozotocin (STZ) in citrate buffer solution at a dose of 90 mg/kg. New born rats less than of 48 hr age weighing 7 gm were chosen for the procedure. Experiments were conducted three months after the STZ injection. The type 2 diabetic rats were selected for the experiment after conducting an oral glucose tolerance test (OGTT) and only the diabetic model rats with blood glucose levels of 8–12 mmol/L under fasting conditions were selected for the experiments as previously described [21, 22].

### 2.4. Chronic and Acute Effects of Ethanolic Extract of* B. monosperma* on Glucose Homeostasis

To evaluate effects on fasting blood glucose, the* B. monosperma* extract (250 mg/kg, 500 mg/kg and 1000 mg/kg) was suspended in distilled water and orally administered to 12 h fasted type 2 diabetic rats. The control animals received an equal volume of distilled water.

Effects on glucose tolerance were similarly evaluated by administration of* B. monosperma* extracts together with glucose (2.5 g/10 mL per kg body weight) after a fasting period of 12 h. Control group received only glucose solution.

In either cases blood was collected from the tail vein, and serum separated by centrifugation and stored at −22°C until further analysis. Blood glucose was analysed by GOD-PAP method [23] (glucose kit, Randox, UK).

To evaluate chronic effects of* B. monosperma*, type 2 diabetic rats were given extract at 250, 500, and 1000 mg/kg doses by gavage, twice daily for 48 d. Control rats were similarly administered water alone (10 mL/kg body weight). Blood samples were collected from the cut tip of the tail at the times indicated in the figures (Figure 4). Serum was separated by centrifugation, stored, and analyzed as mentioned above.

### 2.5. Effects of* B. monosperma* on Plasma Insulin

Blood was drawn from fasted type 2 diabetic rats, 1 hr after administration of* B. monosperma*. The amount of insulin released from the pancreas in vivo was determined using Rat Insulin ELISA Kit (Crystal Chem, USA).

### 2.6. Effects of* B. monosperma* on Insulin Secretion from Isolated Pancreatic Islets

Pancreatic islets were isolated by collagenase digestion with minor modifications as previously described [24]. The amount of insulin released from the isolated islets in the presence of 3 mM and 11 mM glucose by* B. monosperma* extract at different doses (20, 40, and 80 mg/mL) was determined by Rat Insulin ELISA Kit (Crystal Chem, USA).

### 2.7. Effects of* B. monosperma* on Liver Glycogen Content

Briefly, after the 48 days of chronic administration of the extract to the type 2 diabetic rats, the liver was collected, weighed, and finely homogenized with 20 mL of 5% trichloroacetic acid (TCA). The proteins precipitated, which was filtered off, and the clear filtrate was analysed for glycogen. The liver glycogen content was determined following the anthrone method as described previously [25].

### 2.8. Effects of* B. monosperma* on Intestinal Glucose Absorption

An in situ intestinal perfusion technique [26] was used to determine the effect of* B. monosperma* intestinal absorption of glucose in 36 h fasted nondiabetic rats anaesthetized using Ketamine (80 mg/kg). Ethanol extract of* B. monosperma* (5 mg/mL, 10 mg/mL, and 20 mg/mL equivalent to 0.25 g/kg, 0.5 g/kg, and 1 g/kg) was suspended in Krebs Ringer buffer, along with glucose (54 g/L). These were passed through rat pylorus via a butterfly cannula and the perfusate collected by means of a tube inserted at the end of ileum. The control group was perfused with Krebs Ringer buffer along with glucose only. Perfusion was carried out at a rate of 0.5 mL/min for 30 min at 37°C. The results were presented as percentage of absorbed glucose, calculated from the percentage change in the amount of glucose in solution before and after the perfusion.

### 2.9. Effects of* B. monosperma* on Sucrose Absorption from the Gastrointestinal Tract

The effect of* B. monosperma* on sucrose absorption from gastrointestinal tract was assayed by determining the unabsorbed sucrose content following oral sucrose load by six-segment study as described by Hannan et al. [27]. 12 h fasted type 2 diabetic rats were administered 50% sucrose solution per oral (2.5 g/kg body mass) along with three doses of* B. monosperma* (250 mg/kg, 500 mg/kg, and 1000 mg/kg) and equal volume of water for control. Blood was sampled at the following time intervals: 30, 60, 120, and 240 min, after sucrose load for the quantification of blood glucose. At these time intervals, some of the rats were sacrificed for determining unabsorbed sucrose contents of the GI tract. The GI tract was excised and separated into six segments: the stomach, the upper 20 cm, middle and lower 20 cm of the small intestine, the caecum, and the large intestine. Each segment was rinsed with acidified ice-cold saline followed by centrifugation at 3000 rpm (1000 g) for 10 min. The supernatant was pipette out and boiled for 2 h, in sulphuric acid, to hydrolyse the sucrose. The sulfuric acid was later neutralized by NaOH solution. Both plasma glucose concentration and the amount of glucose released from residual sucrose in the GI tract were determined. The GI sucrose content was calculated from the amount of liberated glucose [28].

### 2.10. Effects of* B. monosperma *on Gut Motility

Gastrointestinal motility was determined by means of BaSO_4_ milk following the previously described method of Chatterjee [29]. BaSO_4_ milk was prepared by mixing BaSO_4_ as 10% (w/v) in 0.5% carboxymethyl cellulose to form a suspension. The ethanol extract was administered per oral, 1 hr before the oral administration of BaSO_4_ milk. Control group was administered distilled water only (10 mL/kg). Rats belonging to all groups were sacrificed 15 min after BaSO_4_ administration. The distance travelled by BaSO_4_ milk was measured and represented as a percentage of total length of the small intestine (from pylorus to ileocaecal junction).

### 2.11. Effects of* B. monosperma* on Intestinal Disaccharidase Enzyme Activity

The assay was conducted following the procedure as described previously by Hannan et al. [27]. The ethanol extract of* B. monosperma* (250, 500, and 1000 mg/kg) was administered by gastric gavage to 20 hr fasted nondiabetic rats. After 60 min, the rats were sacrificed and the small intestine was isolated, cut longitudinally, rinsed with ice-cold saline, and homogenized in 10 mL saline (0.9% NaCl). Aliquots of homogenate were incubated with 40 mM sucrose at 37°C for 60 min. The amount of protein was determined by DC Protein Kit (Bio Rad, USA). Disaccharidase activity was determined from the glucose concentration converted from sucrose as *μ*mol/mg protein/h.

### 2.12. Effect of* B. monosperma* on Body Mass, Food, and Water Intake of Type 2 Diabetic Rats

The rats kept for chronic study were provided with sufficient amounts of food and water for one day. At the end of the day, the mass of food and volume of water intake was recorded. The change in body mass of the rats was also monitored at periods as shown in the graph (Figure 9).

### 2.13. Effects of* B. monosperma* on Organ Weight Ratio of Liver and Pancreas

The animals used on the chronic study were sacrificed by cervical dislocation at the end of the study period and the liver and pancreas were excised. They were cleaned of fats and were kept moist at all times keeping them in normal saline (0.9% of NaCl). The wet mass of the organs was immediately weighed using a digital balance. The weight of the pancreas was expressed as mg/100 gm of body weight, while the weight of the liver was represented as gm/100 gm of body weight, as shown in the graphs (Figure 10).

### 2.14. Chronic Effects of Ethanolic Extracts of* B. monosperma* on Serum Lipid Profile of Type 2 Diabetic Model Rats

To assess chronic effects of* B. monosperma*, type 2 diabetic model rats were treated with ethanol extract of* B. monosperma* at three dosages (250 mg/kg, 500 mg/kg, and 1000 mg/kg) by gastric gavages, twice daily for 48 d. Control rats were administered only distilled water of similar volume. Blood samples were collected from the tail vein, at times, indicated in the graphs (Figure 11). Serum was separated by centrifugation and stored at 22°C until further analysis.

### 2.15. Effect of* B. monosperma* on Jejunal Nutrient Absorption by Glucose Dialysis-Tube Retardation Assay

Dry, precut dialysis sacs (inflated diam. approx. 16 mm, length = 30 cm, Sigma Aldrich, USA) were soaked in 1 g sodium azide/L. The bag was loaded with 6 mL sodium azide (1 gm/L) and 36 mg glucose alone (the control sac) or after addition of fine powder of* B. monosperma*. The dry fibrous powder was wetted by an aqueous solution of sodium azide (1 g/L) for 14 h prior to the experiment. The sacs were closed at the ends and hung in a solution of 100 mL of sodium azide (1 g/L) and then placed in a stirred bath at 37°C for 1 hr. At 30 and 60 min time interval, 2 mL of the dialysate was analyzed for glucose by the GOD-PAP method following the manufacturer's instruction manual available in the website of Sigma Aldrich.

The effect of fiber on nutrient absorption was indicated by the glucose dialysis retardation index:

(1) −Total  glucose  diffused  from  sac  containing  fiberTotal  glucose  diffused  from  sac  containing  no  fiber  present×100.

### 2.16. Effect of* B. monosperma* on *α*-Amylase Activity

The effects of* B. monosperma* powder on starch digestibility were determined as a function of time in a fiber-enzyme-starch mixture system using a dialysis membrane with a cutoff molecular weight of 12,000 da (inflated diam. approx. 16 mm, length = 30 cm, Sigma Aldrich, USA) as previously described with minor modifications [30]. A solution was prepared by mixing 0.2 g of powdered* B. monosperma* and 0.04 g *α*-amylase (obtained from human saliva, Sigma Aldrich, USA) in 10 mL of potato starch solution (4 g/100 mL) was dialyzed in 200 mL deionised water at 37°C. Following the incubation period, 10, 30, 60, and 120 min, glucose concentration in the dialysate solution was assayed using the GOD-PAP method as described previously. The control was run without the addition of powder.

### 2.17. Determination of Glucose-Adsorption Capacity

The assay was conducted following the procedure by Ou et al. [31], where the glucose-adsorption ability (mM/mol/gm) was measured by mixing 1 g of insoluble plant powder or carboxymethyl cellulose (CMC) with 100 mL of glucose solution at a constant temperature of 37°C for 6 hr. This was then followed by centrifugation at 3500 rpm for 15 min. Glucose concentration in the supernatant was assayed using GOD-PAP method as previously described.

### 2.18. Statistical Analysis

Statistical tests were conducted using Statistical Package for Social Science Software (SPSS) ver. 20 (IBM, Inc., Chicago, IL, USA). Results are presented as means ± SEM or mean ± SD, whichever appropriate. Data from experimental groups were compared using unpaired Student's* t*-test and the Mann-Whitney* U*-test, as required. Experiments with data being collected at several time intervals were analyzed using repeated measures ANOVA followed by Bonferroni adjustment ensuring an error margin within ≤5%. One-way ANOVA was carried out and pair-wise comparisons were made with the control group using Dunnett's test to maintain an acceptable error margin of 5%. A two-tailed *P* value of <0.05 was considered statistically significant.

## 3. Results

### 3.1. Acute and Chronic Effects of* B. monosperma* on Glucose Homeostasis

**Figure 1:**
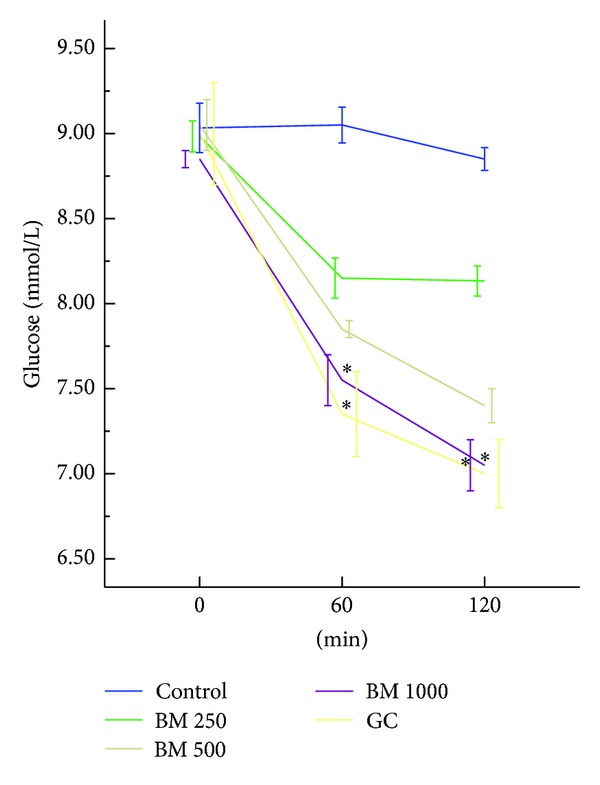
Effects of ethanol extract of* Butea monosperma *(BM) on fasting blood glucose (FBG) level in type 2 diabetic rats. Values are means and standard deviations represented by vertical bars (*n* = 10). Fasted rats were given ethanol extract of BM (250 mg/kg, 500 mg/kg, and 1000 mg/kg body weight) or Glibenclamide (GC) (0.5 mg/Kg) by oral administration. Mean values marked with an asterisk (∗) were significantly different from those of respective control rats (*P* < 0.05) (derived from repeated measures ANOVA and adjusted using Bonferroni correction).

**Figure 2:**
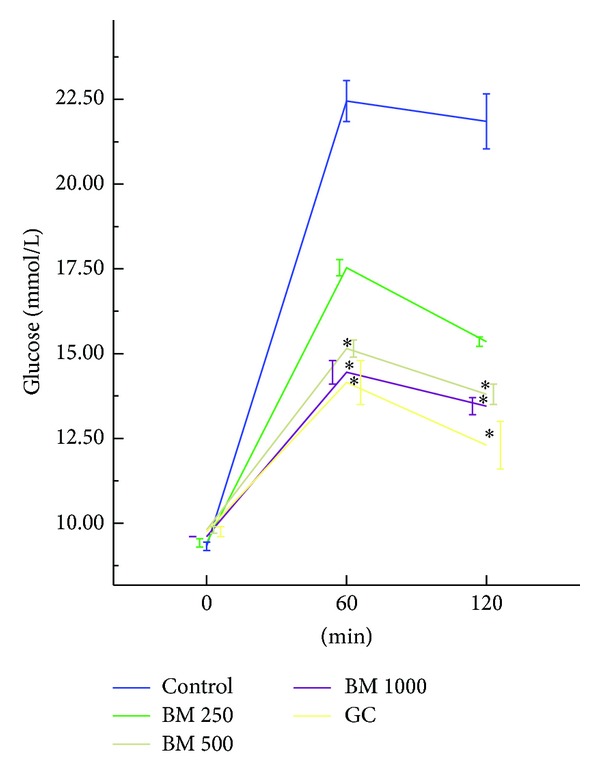
Effects of ethanol extract of* Butea monosperma* (BM) on oral glucose tolerance test (OGTT) in type 2 diabetic rats. Values are means and standard deviations represented by vertical bars (*n* = 11). Fasted rats were given ethanol extract of BM (250 mg/kg, 500 mg/kg, and 1000 mg/kg body weight) or Glibenclamide (GC) (0.5 mg/Kg) by oral administration with glucose (2.5 g/kg body weight). Mean values marked with an asterisk (∗) were significantly different from those of respective control rats (*P* < 0.05) (derived from repeated measures ANOVA and adjusted using Bonferroni correction).

**Figure 3:**
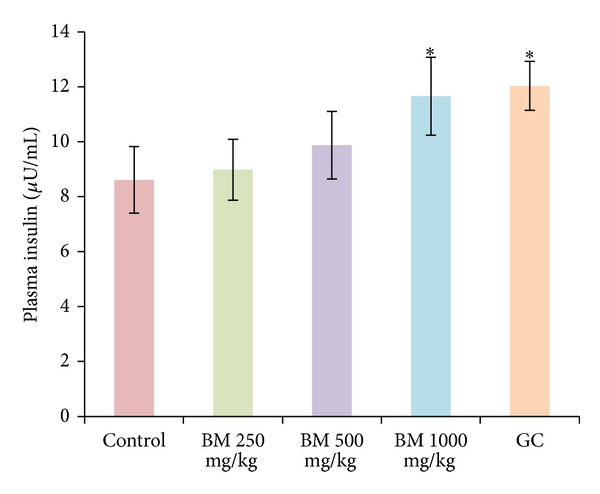
Effects of ethanol extract of* Butea monosperma* (BM) on serum insulin level in fasted type 2 diabetic rats. Values are means and standard deviations represented by vertical bars (*n* = 8). Rats were given ethanol extract of BM (250 mg/kg, 500 mg/kg, and 1000 mg/kg body weight) or Glibenclamide (GC) (0.5 mg/Kg) by oral administration. Mean values marked with an asterisk (∗) were significantly different from those of respective control rats (*P* < 0.01) (derived from repeated measures ANOVA and adjusted using Bonferroni correction).

Oral administration of* B. monosperma*, in a dose-dependent manner, changed the antihyperglycemic condition of fasted type 2 diabetic rats; however, only the effect of 1000 mg/kg dose was significant (*P* < 0.05) (Figure 1). The extract, at 500 mg/Kg and 1000 mg/Kg doses, improved glucose homeostasis both at 60 min and 120 min, when administered along with glucose load (*P* < 0.05; Figure 2). The extract also increased the level of plasma insulin significantly at 1000 mg/Kg dose (*P* < 0.01; Figure 3).

**Figure 4:**
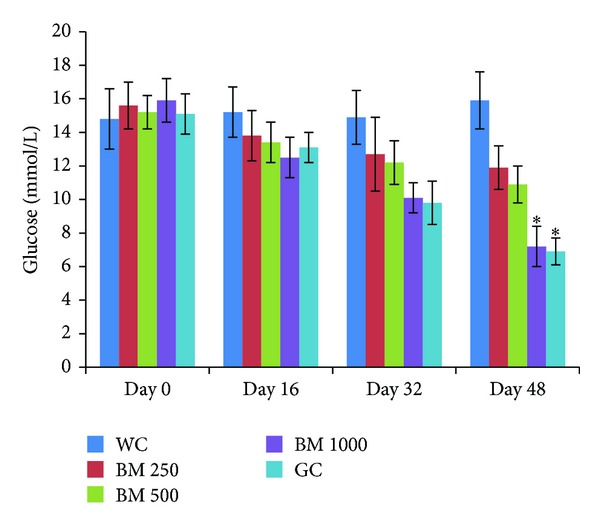
Effects of ethanol extract of* Butea monosperma* (BM) on fasting serum glucose level in type 2 diabetic rats after 48 days of feeding. Values are means and standard deviations represented by vertical bars (*n* = 10). Fasted rats were given ethanol extract of BM (250 mg/kg, 500 mg/kg, and 1000 mg/kg body weight) or Glibenclamide (GC) (0.5 mg/Kg) by oral administration for a period of 48 days. Mean values marked with an asterisk (∗) were significantly different from those of respective control rats (Water control, WC) (*P* < 0.05) (derived from repeated measures ANOVA and adjusted using Bonferroni correction).

After a 48 days chronic study of* B. monosperma* (three doses, administered twice daily) on type 2 diabetic rats, both 500 mg/Kg and 1000 mg/Kg doses showed significant reduction in serum glucose level (*P* < 0.05; Figure 4).

### 3.2. Effect of* B. monosperma* on Serum Glucose after Sucrose Load

**Figure 5:**
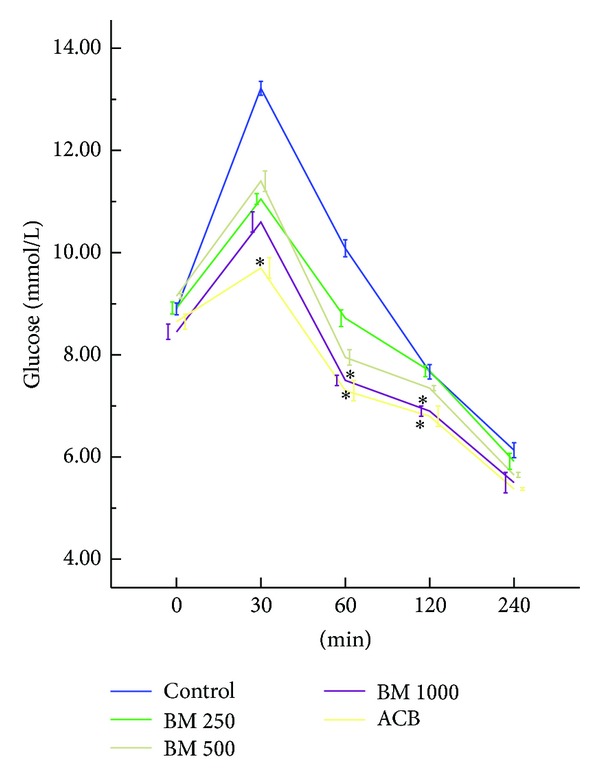
Effects of ethanol extract of* Butea monosperma* (BM) on serum glucose after the sucrose load in type 2 diabetic rats. Rats were fasted for 20 h and administered orally with a sucrose solution (2.5 g/kg body weight) with or without ethanol extract of BM (250 mg/kg, 500 mg/kg, and 1000 mg/kg body weight) or Acarbose (ACB) (200 mg/Kg). Values are means and standard deviations represented by vertical bars (*n* = 8). Mean values marked with an asterisk (∗) were significantly different from those of respective control rats (*P* < 0.05) (derived from repeated measures ANOVA and adjusted using Bonferroni correction).

Only the 1000 mg/Kg dose of* B. monosperma* showed a significant (*P* < 0.05) suppression of serum glucose level at 60 min and 120 min compared to control, where a quick peak serum glucose was observed after administration of sucrose load (Figure 5).

### 3.3. Effect of* B. monosperma* on Intestinal Glucose Absorption

**Figure 6:**
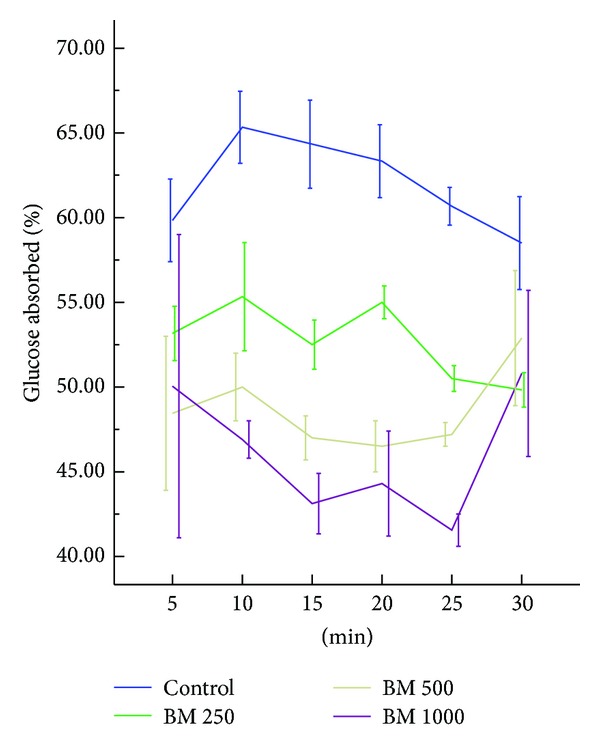
Effects of ethanol extract of* Butea monosperma* (BM) on intestinal glucose absorption in type 2 diabetic rats. Rats were fasted for 36 h and the intestine was perfused with glucose (54 g/L) with or without ethanol extract of BM (5 mg/mL, 10 mg/mL, and 20 mg/mL; each subject received 15 mL of perfusion). Values are means and standard deviations represented by vertical bars (*n* = 9). Mean values marked with an asterisk (∗) were significantly different from those of respective control rats (*P* < 0.05) (derived from repeated measures ANOVA and adjusted using Bonferroni correction).

*B. monosperma* extract, when was perfused with glucose, failed to show any significant reduction in the extent of glucose absorption during the perfusion period (Figure 6).

### 3.4. Effect of* B. monosperma* on Unabsorbed Sucrose Content in the Gastrointestinal Tract

**Figure 7:**
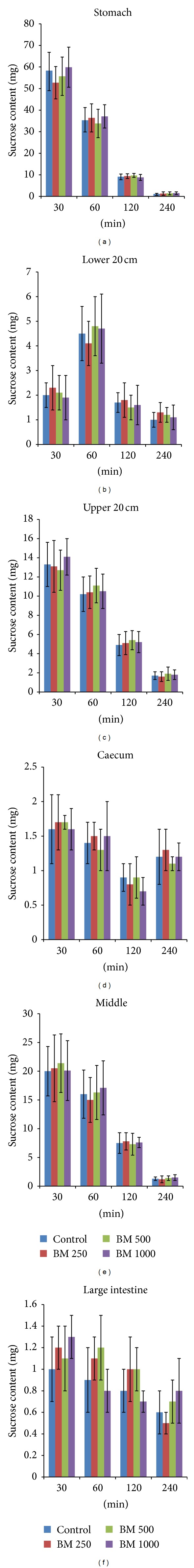
Effects of ethanol extract of* Butea monosperma* (BM) on gastrointestinal sucrose content after oral sucrose loading in type 2 diabetic rats. Rats were fasted for 20 h before the oral administration of a sucrose solution (2.5 g/kg body weight) with or without ethanol extract of BM (250 mg/kg, 500 mg/kg, and 1000 mg/kg body weight). Values are means and standard deviations represented by vertical bars (*n* = 8). Mean values marked with an asterisk (∗) were significantly different from those of control rats (*P* < 0.05) (derived from repeated measures ANOVA and adjusted using Bonferroni correction).

Upon oral administration of sucrose along with* B. monosperma* (1000 mg/Kg), there was no significant amount of unabsorbed sucrose present in the stomach, upper, middle, lower intestine, caecum, and large intestine (Figure 7).

### 3.5. Effect of* B. monosperma* on Gut Motility and Intestinal Disaccharidase Enzyme Activity

**Figure 8:**
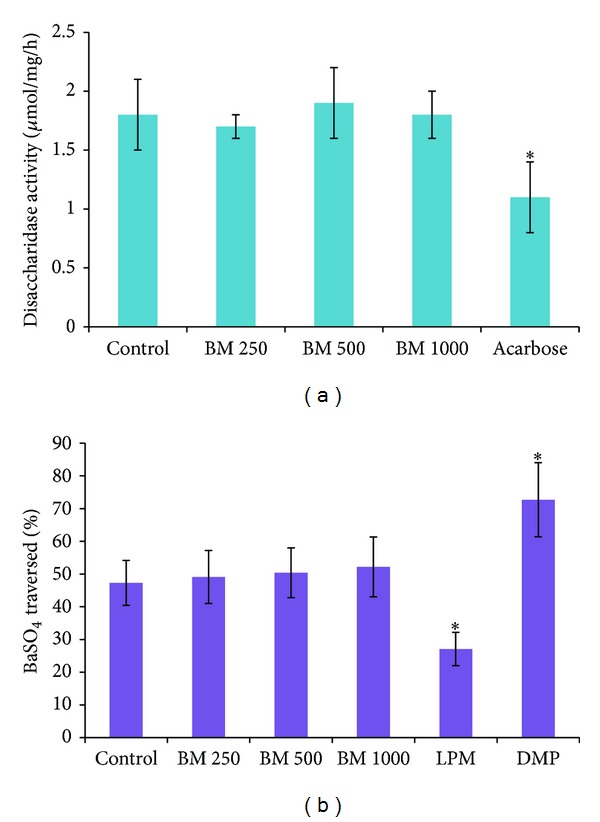
Effects of ethanol extract of* Butea monosperma* (BM) on (a) intestinal disaccharidase activity and (b) gastrointestinal motility (by BaSO_4_ traversed) in type 2 diabetic rats. Rats were fasted for 20 h before the oral administration of ethanol extract of BM (250 mg/kg, 500 mg/kg, and 1000 mg/kg body weight). Enzyme activity was determined and BaSO_4_ was administered at 60 min. Motility was measured over the following 15 min. Acarbose (200 mg/Kg) and Loperamide (LPM) 5 mg/Kg) and Domperidone (DMP) (10 mg/Kg) were used as reference controls for disaccharidase activity test and gastrointestinal motility test respectively. Values are means and standard deviations represented by vertical bars (*n* = 12). Mean values marked with an asterisk (∗) were significantly different from those of diabetic control rats (*P* < 0.05) (derived from repeated measures ANOVA and adjusted using Bonferroni correction).

None of the doses of* B. monosperma* extract altered the gastrointestinal motility. The extract also failed to show any significant inhibition of disaccharidase enzyme activity (Figure 8).

### 3.6. Effect of* B. monosperma* on Insulin Secretion from Isolated Rat Islets

*B. monosperma* extract, at 80 *μ*g/mL dose, stimulated insulin secretion significantly from the isolated rat islets in the presence of both 3 mM and 11 mM glucose (Table 1).

### 3.7. Chronic Effect of* B. monosperma* on Liver Glycogen, Organ Weight, Food Habit, and Serum Lipid Profile

**Figure 9:**
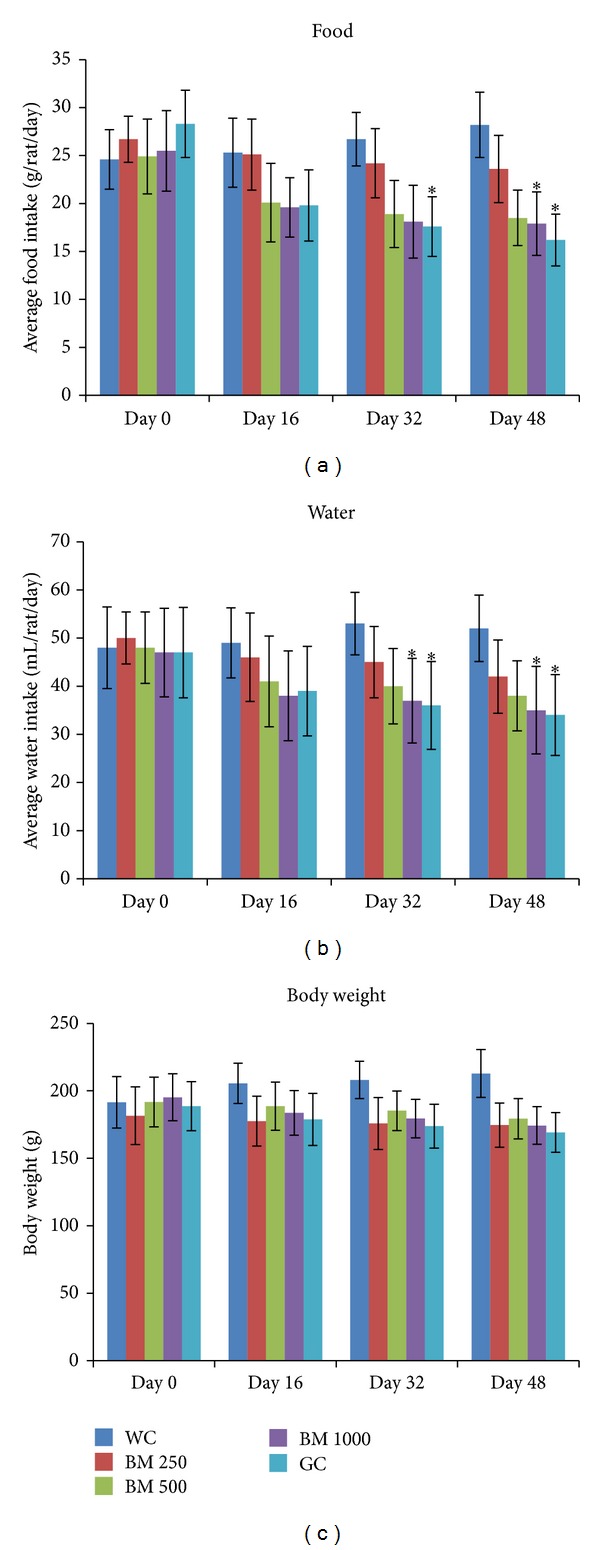
Effects of ethanol extract of* Butea monosperma* (BM) on body weight, average food intake, and average water intake in type 2 diabetic rats after 48 days of feeding. Values are means and standard deviations represented by vertical bars (*n* = 10). Fasted rats were given ethanol extract of BM (250 mg/kg, 500 mg/kg, and 1000 mg/kg body weight) or Glibenclamide (GC) (0.5 mg/Kg) by oral administration for a period of 48 days. Mean values marked with an asterisk (∗) were significantly different from those of respective control rats (Water control, WC) (*P* < 0.05) (derived from repeated measures ANOVA and adjusted using Bonferroni correction).

**Figure 10:**
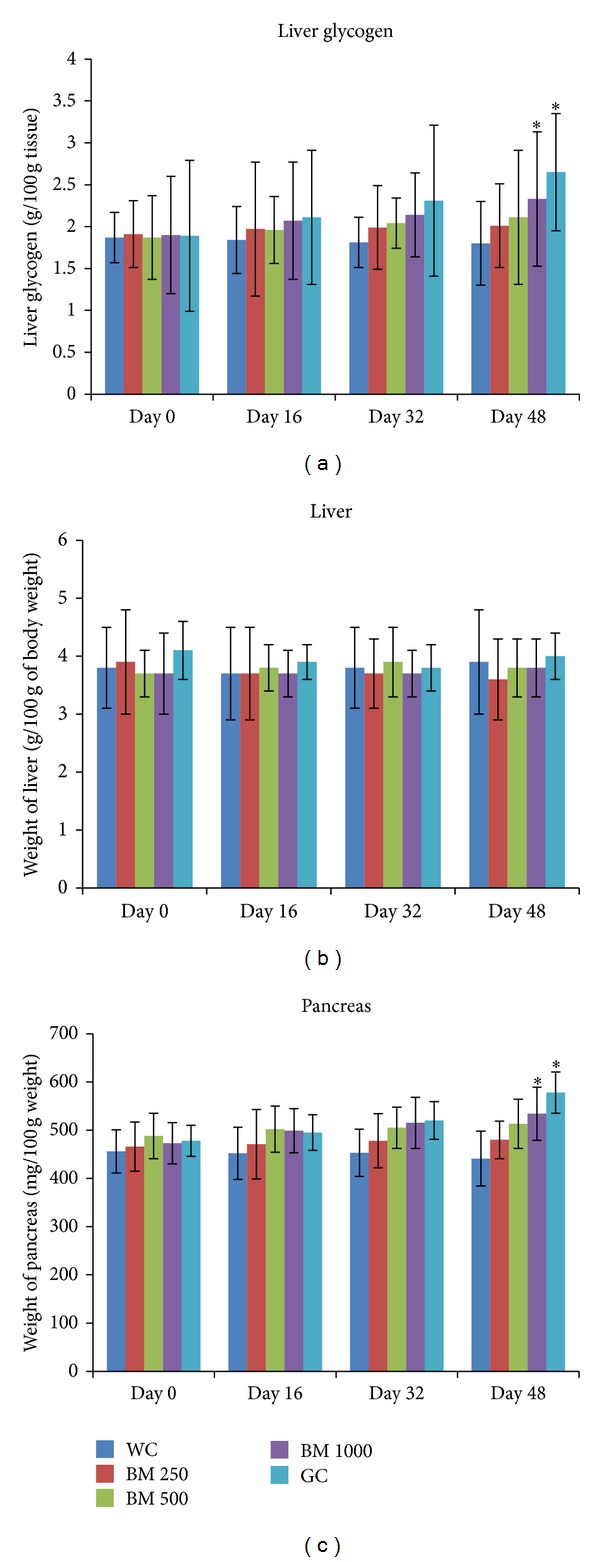
Effects of ethanol extract of* Butea monosperma* (BM) on liver glycogen, liver weight, and pancreas weight in type 2 diabetic rats after 48 days of feeding. Values are means and standard deviations represented by vertical bars (*n* = 10). Fasted rats were given ethanol extract of BM (250 mg/kg, 500 mg/kg, and 1000 mg/kg body weight) or Glibenclamide (GC) (0.5 mg/Kg) by oral administration for a period of 48 days. Mean values marked with an asterisk (∗) were significantly different from those of respective control rats (Water control, WC) (*P* < 0.05) (derived from repeated measures ANOVA and adjusted using Bonferroni correction).

After the 48 days long study of* B. monosperma* (three doses: 250, 500, and 1000 mg/Kg, administered twice daily) on type 2 diabetic rats, significant reduction in food intake, and water intake was observed (*P* < 0.05; Figure 9). However, no significant change was seen in overall body weight. The chronic treatment of the extract also significantly increased liver glycogen content and pancreas weight; however, no change in liver weight was observed (*P* < 0.05; Figure 10).

**Figure 11:**
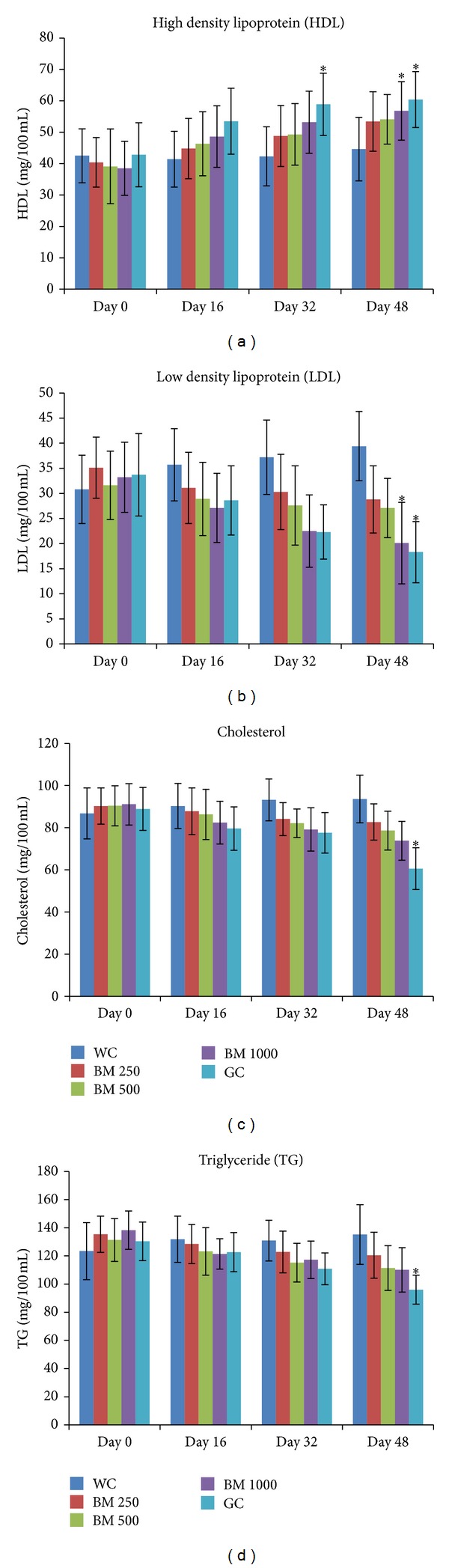
Effects of ethanol extract of* Butea monosperma* (BM) on serum lipid profile (TG, Cholesterol, HDL, and LDL) in type 2 diabetic rats after 48 days of feeding. Values are means and standard deviations represented by vertical bars (*n* = 10). Fasted rats were given ethanol extract of BM (250 mg/kg, 500 mg/kg, and 1000 mg/kg body weight) or Glibenclamide (GC) (0.5 mg/Kg) by oral administration for a period of 48 days. Mean values marked with an asterisk (∗) were significantly different from those of respective control rats (Water control, WC) (*P* < 0.05) (derived from repeated measures ANOVA and adjusted using Bonferroni correction).

1000 mg/Kg dose of* B. monosperma* extract improved serum lipid profile of type 2 diabetic rats after 48 days of twice daily oral feeding. It decreased the level of serum triglyceride, low density lipoprotein (LDL), and cholesterol significantly, which was comparable to the effect of reference drug Glibenclamide. Moreover, the extract increased the level of high density lipoprotein (HDL) significantly (*P* < 0.05; Figure 11).

### 3.8. Effect of* B. monosperma* Powder on In Vitro Glucose Dialysis Retardation Index (GDRI)

*B. monosperma* powder did not significantly reduce the amount of glucose present in the dialysate compared to the control group (Table 2).

### 3.9. Effect of* B. monosperma* Powder on In Vitro Glucose Adsorption Capacity

*B. monosperma* powder failed to show high capacity of glucose adsorption in the presence of different levels of glucose in the solution (Table 3).

### 3.10. Effect of* B. monosperma* Powder on *α*-Amylase Activity

No effect of* B. monosperma* powder on starch digestibility was found by the alteration in the glucose concentration in the dialysate with time. There was no significant change, compared to control, in the glucose content at 10 min to 120 min (Table 4).

## 4. Discussion

Our study incorporates a wide range of methods to fully understand the possible underlying mechanism of antidiabetic action of* B. monosperma*. Preliminary studies involving determination of FBG, GTT, chronic blood glucose, and blood glucose after sucrose load, and all showed a marked, dose-dependent decline in blood glucose level on* B. monosperma* administration. These findings corroborated with previous studies on* B. monosperma* claiming antihyperglycemic activity [13–16]. Improved background glucose control hinders progression of diabetic macro- and microvascular diseases [32, 33]. Acute hyperglycemia is known to cause pseudohypoxia. Effects of pseudohypoxia on neuronal and renal functions are mediated by a cascade of imbalances in lipid metabolism, increased production of superoxide ions, and increased nitric oxide formation, all of which are undesirable [34].

Insulin resistance as found in type 2 diabetic patients often results in various of pathological states, including, dyslipidemia, increased activity of endogenous procoagulants, endothelial dysfunction, changes in hemodynamic status, and so forth, all of which increase the risk of cardiovascular diseases [35, 36]. The dyslipidemic condition found concurrently with insulin resistance and type 2 diabetes is characterized by increased level of serum triglycerides (TG) and decreased serum HDL [37–39]. Type 2 diabetic individuals have a 2–4 times higher risk of cardiovascular diseases when compared to their nondiabetic counterparts. Chronic dyslipidemic state is thought to be the main factor of the increased risk [40]. On chronic study, type 2 diabetic rats treated with the* B. monosperma* showed a marked reduction in serum LDL, cholesterol, and TG level and a significant improvement in serum HDL level. Active compounds from* B. monosperma* therefore can be further explored to combat dyslipidemia associated with type 2 diabetes. Existing data on lipid profile of* B. monosperma* treated type 2 diabetic rats further substantiates our findings [13–16].

Other parameters observed during the chronic study were the liver, pancreas, and body weight. The food and water intake of the test animals were also monitored. Both food and water intake of* B. monosperma* treated type 2 diabetic rats decreased. This might be taken as a preliminary indication of improvement of their diabetic condition, as type 2 diabetic rats typically experience both polydipsia and polyphagia [41]. Changes in body weight and weight of the liver were insignificant throughout the test groups. Weight of the pancreas, however, significantly increased. Glibenclamide and its analogues are known to be capable of increasing the beta cell mass [42] and its proliferation in pancreatic islets [43]. However, our observation regarding increased pancreatic mass is still too preliminary to draw any conclusion with.

Several investigators have elucidated ability of complex carbohydrate, high molecular weight, and viscous, soluble dietary fibers, to retard glucose absorption [44–46]. Due to its high viscosity and fibre binding ability, dietary fibers, at times, proves to significantly retard absorption [44]. Dietary fibers are capable of significantly reducing the transit time in GI tract of ingested food [47]. Reduced transit time results in shorter period available for the carbohydrates in the meal to be digested and absorbed [48]. It is therefore a valuable mode of preventing postprandial hyperglycemia but has not been extensively studies till to-date. In our studies, however, both the in vitro assays, meant to study,* B. monosperma* fibre-glucose binding yielded no significant results. This was further confirmed by carrying out a set of two in vivo assays, “perfusion of rat intestine” and “six segments study on isolated rat intestine.” These two assays, in conjunction, reconfirm our observation in the in vitro tests. Additionally, GI motility test showed no significant change in this parameter. Consequently, the possibility of hindered sugar absorption, being a possible mechanism of activity of* B. monosperma,* can be ruled out.

Reduced carbohydrate digestion is highly effective in controlling postprandial glucose in diabetic patients. Acarbose, an *α*-glucosidase inhibitor specifically reduces postprandial hyperglycemia. Acarbose treatment resulted in a 36% risk reduction of progression to diabetes [49], 34% risk reduction in the development of new cases of hypertension, and 49% risk reduction in cardiovascular events [50]. In vitro enzyme activity assays confirmed the lack of significant inhibitory activity of* B. monosperma* on *α*-amylase and extracted intestinal disaccharidases. Hence,* B. monosperma* probably does not inhibit digestion of starch or other edible disaccharides.

Intensive insulin therapy is invaluable in treatment of type 1 diabetes. Diabetic patients affected by type 2 diabetes on the other hand are usually treated with insulin sensitizers to enhance body's response to the endogenously secreted insulin. Studies, however, have shown that type 2 diabetic patients exposed to higher than normal insulin level experience additional insulin secretion, normalization of hepatic glucose utilization, and partial reversal of the postbinding defect in peripheral insulin action [51]. It is widely acknowledged that abnormality in insulin secretion and action plays significant role in development of type 2 diabetes. “Insulin secretory dysfunction in type 2 diabetes is both qualitative and quantitative,” as stated by Pratley et al. [52]. Type 2 diabetic patients also experience marked reduction in peripheral glucose utilization [53].

The novel and most remarkable findings of our study were elevation of blood insulin level in type 2 diabetic rats, stimulated insulin release from isolated rat islets, and increased deposition of glycogen in rat liver. Active compound or an amalgam active compounds of in* B. monosperma *have strong potential in treating both types 1 and 2 diabetes by enhancing quality and quantity of insulin secreted and by enhancing hepatic glucose utilization. A notable limitation of the current study was a lack of histological analysis, which could have corelated the enhanced insulin secretion to improved beta-cell morphology. We are currently working on isolating potential bioactive compounds from* B. monosperma* and shed light on receptor level mechanism behind its antidiabetic activity.

## 5. Conclusions

A near exhaustive study was carried out on ethanolic extract of* B. monosperma*, where we made an attempt to explore almost every possible dimension through which it might impart its previously claimed antihyperglycemic activity. Our present study rules out possibilities of retarded glucose absorption or inhibition of enzyme activities by* B. monosperma*. A triad of plasma insulin assay stimulated insulin secretion from isolated rat islets and liver glycogen level provides us with sufficient preliminary evidences, which enables us to attribute the antihyperglycemic action of* B. monosperma* to enhanced insulin secretion and enhanced hepatic glycogen formation.

## Figures and Tables

**Table 1:** Effect of ethanolic extract of BM on insulin secretion from isolated rat islets.

Group	Insulin secretion (ng/mg islet protein)	
	Glucose: 3 mM	Glucose: 11 mM
Control	2.99 (2.65–4.27)	5.41 (4.91–9.27)*
BM 20 *μ*g/mL	2.65 (2.23–3.71)	5.19 (4.41–8.72)
BM 40 *μ*g/mL	3.45 (2.67–3.99)	5.31 (4.18–7.67)
BM 80 *μ*g/mL	5.28 (3.87–6.09)*	7.84 (6.98–9.54)*
Glibenclamide (10 *μ*g/L)	5.89 (4.34–6.95)*	8.92 (7.67–9.86)*

Isolated rat islets were incubated for 60 min with ethanol extract BM (10, 20, and 30 mg/mL) in the presence of 3 or 11 mM glucose. Data are presented as median (range), *n* = 5. Mann-Whitney *U*-test was used to evaluate statistical significance. *P < 0.05 compared with control (3 mM glucose without extract).

**Table 2:** Retarding effect of insoluble fibre of BM on the glucose movement (glucose dialysis retardation index).

Treatment	Dialysis for 30 min		Dialysis for 60 min	
	Glucose in dialysate (mmol/L)	Glucose dialysis retardation index (%)	Glucose in dialysate (mmol/L)	Glucose dialysis retardation index (%)
BM 250 mg	0.93 ± 0.23	21.85	1.55 ± 0.31	9.36
BM 500 mg	0.83 ± 0.33	30.25	1.42 ± 0.29	16.96
BM 1000 mg	0.80 ± 0.12	32.77	1.39 ± 0.42	18.71
CMC 1000 mg	0.62 ± 0.11*	42.86	0.83 ± 0.07*	51.46
Control	1.19 ± 0.21	0	1.71 ± 0.14	0

Data are presented as mean ± SEM (*n* = 7). Glucose dialysis retardation index = control (100%) − fibre (% of control value). Mean values marked with an asterisk (∗) were significantly different from those of respective control groups (*P* < 0.05) (derived from repeated measures ANOVA and adjusted using Bonferroni correction).

**Table 3:** Glucose adsorption capacity of insoluble fibre of BM in different concentrations of glucose.

Treatment	Glucose bound (mmol/g)				
	5 mmol/L	10 mmol/L	50 mmol/L	100 mmol/L	200 mmol/L
BM 250 mg	0.01 ± 0.01^a^	0.26 ± 0.05^a^	2.15 ± 0.51^a^	5.71 ± 1.01^a^	8.39 ± 3.81^a^
BM 500 mg	0.01 ± 0.01^a^	0.71 ± 0.13^a^	2.41 ± 0.87^a^	5.99 ± 1.46^a^	9.10 ± 3.91^a^
BM 1000 mg	0.02 ± 0.01^a^	0.95 ± 0.20^a^	2.89 ± 0.99^a^	6.27 ± 2.02^a^	9.87 ± 4.07^a^
CMC 1000 mg	0.08 ± 0.01^b^	2.71 ± 0.45^b^	9.32 ± 2.09^b^	17.81 ± 3.78^b^	30.97 ± 6.88^b^

Data are presented as mean ± SEM (*n* = 4). Data represent the millimoles of glucose bound by each gram of the BM powdert at different glucose concentrations (5–200 mmol/L). Glucose bound = (glucose concentration of original solution − glucose concentration when the adsorption reached equilibrium) × volume of solution ÷ weight of dietary fibre. Mean values in the same column marked with different letters were significantly different (*P* < 0.05) (derived from repeated measures ANOVA and adjusted using Bonferroni correction).

**Table 4:** Effect of insoluble fibre of BM on starch digestibility.

Treatment	Glucose in dialysate (µmol/L)			
	10 min	30 min	60 min	120 min
BM 250 mg	1.89 ± 0.11	3.99 ± 0.17	11.78 ± 1.55	19.91 ± 4.73
BM 500 mg	1.86 ± 0.15	3.45 ± 0.30	12.61 ± 2.81	19.78 ± 6.11
BM 1000 mg	1.91 ± 0.19	3.48 ± 0.39	12.73 ± 2.73	20.88 ± 5.12
CMC 1000 mg	1.91 ± 0.21	4.94 ± 0.34*	16.09 ± 2.31*	26.57 ± 5.34*
Control	1.52 ± 0.06	3.87 ± 0.12	12.56 ± 1.23	19.51 ± 3.73

Data are presented as mean ± SEM (*n* = 4). Values represent the glucose concentration (µmol) in dialysate. Mean values marked with an asterisk (*) were significantly different from those of respective control groups (*P* < 0.05) (derived from repeated measures ANOVA and adjusted using Bonferroni correction).
